# The effect of local injection of tranexamic acid into peri-articular tissue versus drain clamping in total knee arthroplasty: a randomized controlled trial

**DOI:** 10.1186/s12891-022-05058-6

**Published:** 2022-02-02

**Authors:** Ryosuke Hishimura, Tomohiro Onodera, Yasumitsu Ohkoshi, Kazufumi Okada, Masatake Matsuoka, Shinji Matsubara, Koji Iwasaki, Eiji Kondo, Norimasa Iwasaki

**Affiliations:** 1 Department of Orthopaedic Surgery, Faculty of Medicine and Graduate School of Medicine, Hokkaido University, North 15 West 7, Kita-Ku, Sapporo, 060-8638 Japan; 2 Department of Orthopaedic Surgery, Hakodate Orthopedic Clinic, 2-115, Hakodate, Ishikawa 048-0802 Japan; 3 Clinical Research and Medical Innovation Center, Hokkaido University Hospital, North 15 West 7, Kita-Ku, Sapporo, 060-8638 Japan; 4 Department of functional reconstruction for the knee joint, Hokkaido University, North 15, West 7, Kita-ku, Sapporo, Hokkaido 060-8638 Japan; 5 Centre for Sports Medicine, Hokkaido University Hospital, North 15 West 7, Kita-Ku, Sapporo, 060-8638 Japan

**Keywords:** Tranexamic acid (TXA), Periarticular local injection, Total knee arthroplasty (TKA), Drain clamping

## Abstract

**Background:**

Tranexamic acid (TXA) is used as a synthetic anti-fibrinolytic agent for total knee arthroplasty (TKA) to reduce postoperative bleeding. Though the effects on bleeding reduction of several methods of administering TXA have been demonstrated, the optimal method remains controversial. Recently, the hemostatic effect of periarticular local injection of TXA during TKA was reported. Although this method can be expected to suppress postoperative bleeding without placing a drain, its hemostatic effect has not yet been assessed in comparison with local injection and other methods of administering TXA. The aim of this randomized, prospective study was to assess the efficacy of local injection of TXA during TKA.

**Methods:**

To confirm the effect of the local injection of TXA, drain clamping was set as the control. The subjects included a prospective series of 109 patients randomly divided into 2 groups: the local injection (group L) and the drain clamping (group D). The main outcome measure was postoperative bleeding. Secondary outcomes included pain, physical measurements, and laboratory findings.

**Results:**

The calculated total blood loss (CTBL) in groups L and D was nearly equal and did not show the non-inferiority of group L to group D (883 ± 248 vs. 841 ± 257 ml, *P* = .564). Drained blood loss was significantly higher in group L than in group D (395 ± 130 vs 276 ± 78.8 ml, *P* < .0001). There was no significant difference in hidden blood loss between the groups (488 ± 269 vs 565 ± 261 ml, *P* = .131). The other laboratory findings and physical measurements were identical between the groups.

**Conclusions:**

Although CTBL in group L did not show non-inferiority to group D, the local injection of TXA was considered to be superior for suppressing bleeding considering the risk of the adverse effects of using a drain.

**Trial registration:**

This was a randomized, prospective study registered with UMIN Clinical Trials Registry (Registration number: UMIN000036146, date of disclosure: 10/3/2019).

## Background

Total knee arthroplasty (TKA) is a common surgical procedure that improves activities of daily living in osteoarthritis and rheumatoid arthritis patients, but the clinical results, including postoperative satisfaction, are still insufficient [1, 2]. One of the reasons is postoperative bleeding resulting in pain and restriction of range of motion due to joint fibrosis. Although various hemostatic methods such as use of fibrin sealant [3], cryotherapy [4–6], and tranexamic acid (TXA) [7, 8] have been used to suppress postoperative bleeding after TKA, the most suitable method has not been established.

Administration of TXA is one of the most frequent techniques to prevent bleeding after TKA, and there are several administration routes [7, 9–13]. The systemic administration of TXA by the oral or intravenous route is the most commonly used, and it can significantly reduce blood loss, but it may also be associated with systemic adverse effects, such as nausea, intraoperative hypotension, and venous thromboembolism (VTE) [14, 15]. The other is topical administration, such as drain clamping and intra-articular injection [16–19]. Drain clamping was used as a common method of reducing postoperative bleeding whereby TXA was injected through a drain tube after wound closure, and the tube was then clamped for a few hours [16–18]. Seo et al. reported that the topical administration of TXA minimized systemic complications compared to intravenous administration [20]. The combination of topical administration of TXA and drain clamping is one of the local TXA administration methods. According to the previous studies, the incidence of VTE was almost identical between drain clamping alone and the combination method [17, 18]. This combination therapy could also be expected to show a superior effect on bleeding reduction compared to single administration [19], but there have also been some concerns about disadvantages associated with this method. The American Academy of Orthopaedic Surgeons (AAOS) 2015 clinical practice guideline recommends not using a drain with TKA because of no difference in complications or outcomes [21]. Moreover, the potential risk of retrograde infection has been pointed out [22, 23]. A safe and effective alternative to drain clamping is needed as a hemostatic method.

Local injection of TXA is often used to reduce bleeding in acute trauma surgery [24, 25]. Previous studies suggested that local injection of TXA had a suppressive effect on intra-articular hemorrhage by using a drain [17, 22]. Recently, Yozawa et al. retrospectively confirmed an effect on intra-articular bleeding reduction, which was assessed using a drain, by periarticular injection of TXA during TKA [26]. This method can be expected to suppress postoperative bleeding without placing a drain. Local administration of TXA theoretically reduces systemic adverse effects and avoids the risk of complications due to using a drain. However, whether local TXA is superior to conventional procedures remains controversial. Therefore, we hypothesized that local injection of TXA is non-inferior to drain clamping for reducing postoperative bleeding in TKA. The aim of the present study was to prospectively compare the efficacy of periarticular local injection of TXA without drain clamping to that of injection into the knee joint through a drain combined with drain clamping during TKA.

## Methods

The trial was designed according to the CONSORT guideline. This was a randomized, prospective study registered with the UMIN Clinical Trials Registry (Registration number: UMIN000036146, date of disclosure: 10/3/2019). The protocol of this study was approved by the Hakodate Orthopedic Clinic Institutional Review Board (HOC-2018-C1, 23/7/2018), and the study was performed in accordance with the tenets of the Declaration of Helsinki. The subjects provided their written, informed consent for participation in the study. All methods were carried out in accordance with relevant guidelines and regulations. This research did not receive any specific grant from funding agencies in the public, commercial, or not-for-profit sectors. A total of 120 consecutive patients were scheduled for primary unilateral TKA in Hakodate Orthopedic Clinic. The recruitment start date for the study was September 18, 2018, and the recruitment end date was July 31, 2019. The study was conducted between October 1, 2018 and August 30, 2019. The inclusion and exclusion criteria are detailed in Table 1. Exclusion criteria included the patient underwent extensive synovectomy during surgery due to severe synovitis, use of allogenic blood transfusion postoperatively, and a patient whose drain appeared to be clogged, that is, postoperative volume of drained blood less than 100 ml. In addition, it was decided to stop the study if intraoperative surgical and medical complications, such as intraoperative fracture, neurovascular injury, or myocardial infarction occurred. All eligible patients were randomized into two groups by a clerk who was not involved in medical treatment on the operation day. Randomization into two equal groups was performed with the lottery method just prior to surgery. The operating staff were blinded to the patient assignment until just before implantation during the TKA procedure. TXA was given at 10 mg/kg of body weight. In the local injection group (group L), 25 ml of normal saline containing the above amount of TXA were injected into the area around the incision site, specifically vastus medialis and lateralis, the anterior and posterior capsule, the peripheral part of the resected meniscus, and the synovium. To clarify the role of the local injection in postoperative intra-articular bleeding, drain suction was used in accordance with the previously reported method [26]. Drain suction was started immediately after skin closure. In the drain clamping group (group D), 30 ml of normal saline containing the same amount of TXA were injected after skin closure through a drain. This drainage tube was clamped and closed completely for 120 min, then released.

**Table 1:** Inclusion and Exclusion Criteria

Inclusion Criteria	Exclusion Criteria
Adult over 20 years of age	Sever renal disfunction
Willing and able to give consent	Past history of thrombosis
	Extensive synovectomy during surgery
	Use of the allogenic blood transfusion
	Use of another prothesis due to procedural change
	Patients whose drain was clogged
	Patients who can not prepare the autologous blood

### Surgical technique and perioperative care

All surgical procedures were performed by one surgeon (Y.O.). Under general anesthesia, the air tourniquet was inflated to 280 mmHg during surgery. A midline skin incision was performed with a medial parapatellar approach in all patients. The prosthesis was the posterior-stabilized Persona knee implant system (Zimmer Biomet, Warsaw, IN, USA) in all cases. The bone cementing technique was similar in all cases. No local analgesic cocktail or anti-inflammatory drugs were given in order to elucidate the pharmacological effects of TXA other than hemostasis. A surgical drain was placed into the knee joint. The timing for starting drain suction was different for the two groups, as mentioned above. In both groups, the drains were removed 24 h after surgery. An autologous blood transfusion, which was prepared preoperatively, was performed within 24 h postoperatively.

### Outcome measures

The calculated total blood loss (CTBL) was taken as the primary outcome measure. The CTBL was calculated using a specific formula [27, 28] as the difference between the preoperative and postoperative day (POD) 3 hemoglobin (Hb) level. Secondary outcome measures were as follows. The evaluated blood tests were the volume of drained blood loss (DBL) at postoperative 24 h, hidden blood loss (HBL) on POD 3, and the D-dimer level on POD 7. To evaluate postoperative knee pain, patients were asked to describe their usual knee pain using a numerical rating scale (NRS) on PODs 5 and 10. The NRS is commonly used for the assessment of pain intensity [29, 30], and it is an 11-point scale comprising a number from 0 through 10: 0 indicates “no pain”, and 10 indicates the “worst imaginable pain”. As physical measurements, the range of motion (ROM) on POD 14 and the circumference of the leg at the superior patellar border (suprapatellar girth), 10 cm above the border (thigh girth), and the maximum circumference of the calf (calf girth) on PODs 5 and 10 were measured. Adverse events occurring within POD 14 were also examined. HBL was calculated by subtracting DBL from CTBL, considering that there was almost no bleeding during surgery. The postoperative circumference was evaluated as a percentage based on each preoperative value. The clinical data collection from medical records was performed by an independent blinded observer.

### Statistical analysis

All data are presented as means ± standard deviation (SD). It was assumed that the CTBL could be reduced by 100 ml in group L compared to group D, and that the standard deviation of both groups was 300 ml. From a clinical point of view, the non-inferiority margin was set to 50 ml. From the above settings with a significance level of 0.025 and statistical power of 80%, the sample size was calculated to be 64 patients in each group. A sample size of 72 patients in each group was therefore set under the assumption that 10% of the patients would drop out. Significant differences between two groups were assessed by Student’s *t-*test or the Mann-Whitney U test, as appropriate. Statistical analyses were conducted using JMP Pro version 13.1 and SAS version 9.4 statistical software (SAS Institute, Cary, NC). Significance was accepted with a one-sided *p*-value < 0.025 for the primary outcome measure and with a two-sided *p*-value < 0.05 for the secondary outcome measures.

## Results

**Fig. 1:**
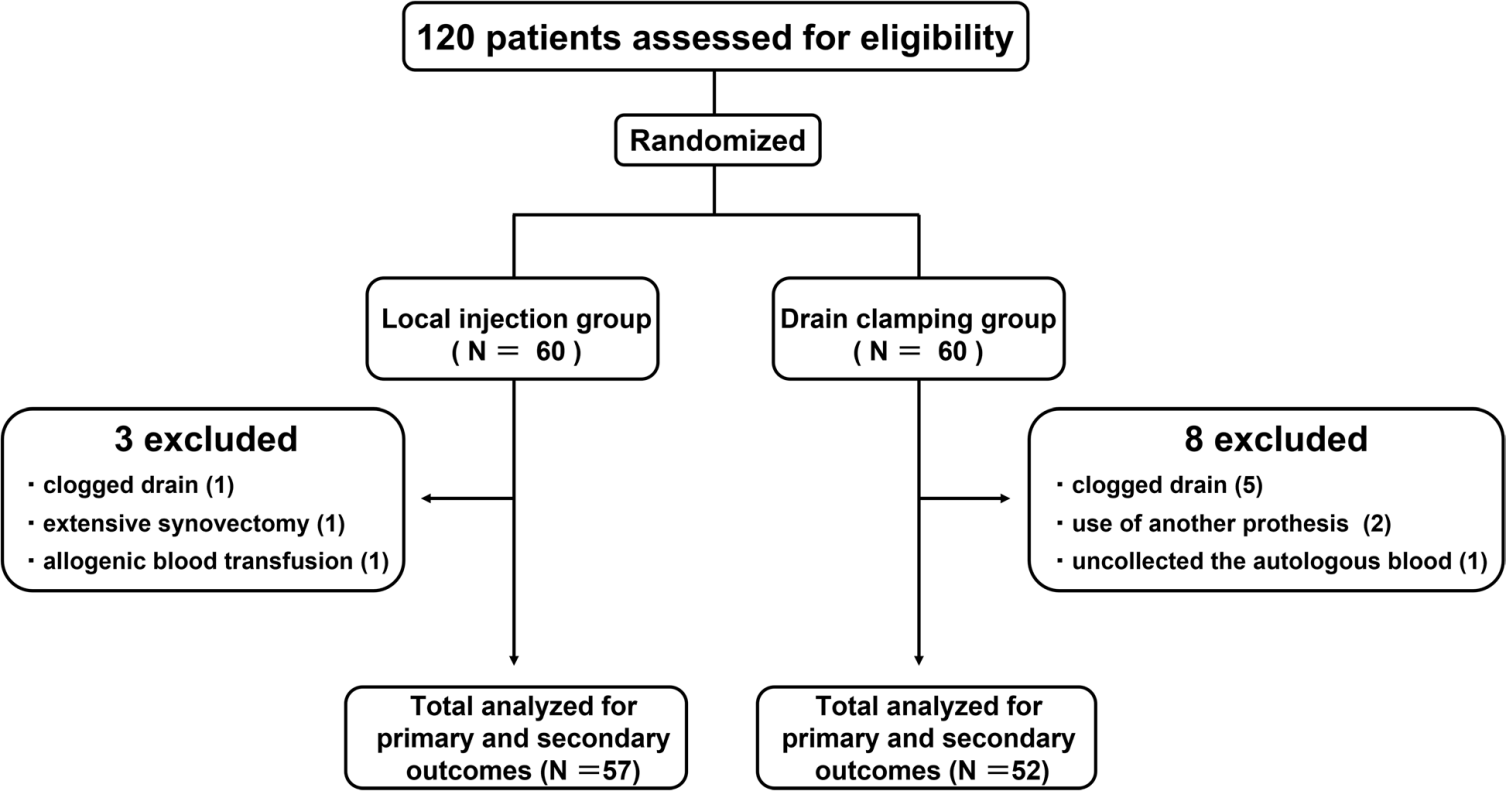
Flow diagram presents the number of patients included through various stages of the study. Eleven patients met the exclusion criteria and 109 patients were included in the analysis

A total of 120 unilateral primary TKAs were prospectively analyzed during the follow-up period. Eleven patients met the exclusion criteria, and 109 patients (57 cases in Group L and 52 cases in Group D) were included in the analysis. The desired sample size was not reached within the study period set by the Ethics Committee. As a result of power recalculation to evaluate its impact, the power was 73.4% with the included sample size. The details of the patients were as follows (Fig. 1): patients whose drain was clogged (6 cases; 1 case in group L and 5 cases in group D); patients for whom autologous blood was not prepared (1 case in group D); patients who underwent extensive synovectomy (1 case in group L); use of allogenic blood transfusion (1 case in group L); and use of another prosthesis (2 cases in group D).

Demographic data of the patients are shown in Table 2. There were no significant differences between the groups in baseline variables.

**Table 2:** Patient Demographics

Variables	Group L	Group D
TKAs	*N* = 57	*N* = 52
Ages (y)	73.0 ± 6.06	72.6 ± 6.07
BMI (kg/m^2^)	27.6 ± 3.85	28.1 ± 3.51
Sex (Female/Male)	49/8	48/4
Diagnosis (OA/others)	54/3	50/2

Values are given as the mean and the standard deviation

OA indicates osteoarthritis, *BMI* Body mass index

The outcomes are summarized in Table 3. As the primary outcome, the CTBL in groups L and D was nearly equal and did not show the non-inferiority of group L to group D (883 ± 248 vs 841 ± 257 ml, *P* = .564 for non-inferiority). While there was no significant difference in HBL between the groups (group L 488 ± 269 ml, group D 565 ± 261 ml, *P* = .131), DBL was significantly higher in group L than in group D (group L 395 ± 130 ml, group D 276 ± 78.8 ml, *P* < .0001). The mean D-dimer level on POD 7 was similar in the two groups (group L 6.50 ± 2.80 μg/ml, group D 5.93 ± 2.71 μg/ml, *P* = .285). The remaining measurements in each group, NRS, ROM, and circumference of the leg, were almost identical (Table 3). No cases of adverse events within POD 14 were reported.

**Table 3:** Postoperative clinical outcomes

Outcomes	Group L	Group D	*P* value
CTBL (ml)	883 ± 248	841 ± 257	0.564
DBL (ml)	395 ± 130	276 ± 78.8	< 0.0001
HBL (ml)	488 ± 269	565 ± 261	0.1315
D-dimer (μg/ml)	6.50 ± 2.80	5.93 ± 2.71	0.285
NRS at POD 5	1.96 ± 2.45	2.65 ± 2.70	0.195
NRS at POD 10	1.81 ± 2.32	2.54 ± 2.56	0.131
ROM at POD 14			
Extension (°)	−0.93 ± 2.32	−1.59 ± 2.92	0.284
Flexion (°)	106 ± 15.1	106 ± 14.3	0.886
Circumference of the leg			
Thigh girth at POD 5 (%)	107 ± 4.80	107 ± 5.25	0.84
Suprapatellar girth at POD 5 (%)	109 ± 4.74	109 ± 4.39	0.556
Calf girth at POD 5 (%)	102 ± 3.96	103 ± 5.11	0.575
Thigh girth at POD 10 (%)	104 ± 4.11	103 ± 4.32	0.77
Suprapatellar girth at POD 10 (%)	107 ± 5.44	106 ± 4.34	0.67
Calf girth at POD 10 (%)	101 ± 4.75	101 ± 7.15	0.79

Values are given as the mean and the standard deviation

CTBL indicates calculated total blood loss. *DBL* Drained blood loss, *HBL* Hidden blood loss, *NRS* Numerical rating scale, *POD* Postoperative day, *ROM* Range of motion

Significance was accepted with a one-sided *p*-value < 0.025 for the CTBL and with a two-sided *p*-value < 0.05 for the secondary outcome measures

## Discussion

The main purpose of this study was to clarify the effect on bleeding reduction of the local injection method using TXA after TKA. The CTBL in the local injection group did not show non-inferiority to that in the drain clamping group. While there was no significant difference in HBL between the groups, DBL was significantly higher in the local injection group than in the drain-clamping group.

Previous retrospective reports showed that drain clamping promoted the tamponade effect, improving intra-articular hemostasis [31, 32]. On the other hand, local injection of TXA significantly reduced the DBL and decreased hemoglobin reduction [33, 34], suggesting that local injection of TXA also had a suppressive effect on intra-articular hemorrhage. The present results showed that the intra-articular hemostatic effect of group D was superior to that of group L, which suggests that the tamponade effect of the injection into the knee joint through a drain of TXA combined with drain clamping exceeds the intra-articular hemostatic effect of the periarticular injection of TXA without drain clamping during TKA. In addition, the DBL of drain clamping with TXA was about 100 ml less, and the standard deviation of the DBL was smaller than in a previous study [18]. This is probably because most of the diseases in this study were OA, and the bleeding risk was relatively small and constant compared to the previous study. On the other hand, clogged drains were seen in 5 group D patients and 1 group L patient. The drain may have been clogged with a hematoma caused by the drain clamping. Although the drain clamping has superior intra-articular hemostasis due to the tamponade effect, the risk of drain clogging should be taken into consideration.

Before starting this study, a non-inferiority trial was designed because we hypothesized that group L had an equivalent effect on bleeding reduction compared to group D. However, non-inferiority of group L compared to group D was not proven for the CTBL. This is at least partly due to the unexpectedly low blood loss in group D. Since the drain clamping method is beneficial for the control of intra-articular hemorrhage, it is better to use it for pathological conditions in which intra-articular hemorrhage is expected to be large. Local injection may be useful for performing wide extra-articular treatment, such as dissection for a knee with contracture.

The results of other laboratory findings and of physical examination (D-dimer, NRS, ROM, and circumference of the leg) were almost identical in the two groups. There are some studies reporting the various effects of TXA other than hemostasis. Several reports indicated that topical administration of TXA does not affect postoperative range of knee motion [20, 35, 36]. The current results showed that the postoperative range of motion was similar in the two groups, which is consistent with previous reports. The animal study showed that TXA could suppress the inflammatory response [37]. Although the anti-inflammatory effect of TXA, resulting in better pain relief and reduced knee swelling, was expected to be stronger with local injection than with intra-articular administration, there were no significant difference in the NRS and the circumference of the leg between the groups. Wong et al. reported that the NRS for postoperative knee pain was almost identical in the TXA and control groups [36]. The present study also showed that there was no significant difference in pain relief and knee swelling according to the method of administration.

Although many orthopedic surgeons placed closed suction drains after TKA empirically, the American Academy of Orthopedic Surgeons (AAOS) clinical practice guideline recommends not using a drain with TKA because of no difference in complications or outcomes. Similarly, randomized, controlled trials and systematic reviews demonstrated no additional benefits of using suction drainage after TKA in terms of patient outcomes [38–41]. The present results demonstrated that the drain clamping method can obtain an excellent reduction in blood loss, including superior intra-articular hemostasis. However, surgeons should pay attention to complications due to drain placement when using the drain clamping method. On the other hand, local injection of TXA has almost the same effect on bleeding reduction without drain placement. This method will be useful as a hemostatic method without the risk of complications associated with drain placement.

In recent years, other hemostatic agents have become more commonly used to control postoperative bleeding [42–45]. A systematic review and meta-analysis suggested that local administration of each hemostatic agent significantly reduced total blood loss during TKA surgery compared with placebo [45]. Systemic administration of TXA is known to carry the risk of adverse effects, including DVT [14, 15]. In the present results, local administration of TXA and the drain-clamping method showed an acceptable hemostatic effect without serious complications.

There were some limitations in this study. First, the assessment period of this study was 14 days after surgery. Although short-term follow-up was considered sufficient to assess the perioperative effects on bleeding reduction of the two different methods, long-term follow-up may result in different clinical outcomes. Second, since the study excluded patients who appeared to be clogged with drainage, the effectiveness of the two hemostatic methods was not fully evaluated. These patients were excluded because one of the objectives was to compare the amount of drainage blood loss (DBL) if the drain worked without clogging. Third, only symptomatic VTE and PE were counted, and D-dimer was evaluated 7 days after surgery in this study. Evaluating asymptomatic VTE and PE using ultrasonography, venography, and chest CT could provide a detailed assessment of the risk of thrombosis. Finally, drain suction was used in this study, whereas the AAOS guidelines do not recommend the use of drains in TKA. Regarding the use of drains in the perioperative period for TKA, regulations differ depending on the country and facility, and a global consensus has not yet been reached. In the present study, the use of a drain facilitated comparison of intra-articular bleeding (DBL) and extra-articular bleeding (HBL) separately between the two groups. Despite its limitations, this study is a meaningful contribution to our understanding of the hemostatic effect of local administration of TXA compared with conventional drain clamping.

## Conclusions

The present results showed that the calculated blood loss in the periarticular local injection of TXA without drain clamping did not show non-inferiority to that in the injection into the knee joint through a drain of TXA combined with drain clamping during TKA. Although one cannot conclude that the local injection method could be an alternative to the drain clamp method in terms of bleeding reduction, local injection of TXA was considered to be a method that can suppress bleeding without injecting fluid into the joint after surgery without drain placement.

## Data Availability

The datasets used and/or analyzed during the current study are available from the corresponding author on reasonable request.

## Abbreviations

TXA: Tranexamic acid
TKA: Total knee arthroplasty
CTBL: Calculated total blood loss
VTE: Venous thromboembolism
AAOS: The American Academy of Orthopaedic
POD: Postoperative day
DBL: Drained blood loss
HBL: Hidden blood loss
NRS: Numerical rating scale
ROM: Range of motion
